# Etiology of Trauma-Related Acute Compartment Syndrome of the Hand: A Systematic Review

**DOI:** 10.7759/cureus.38218

**Published:** 2023-04-27

**Authors:** Obaid Alsaedi, Alwaleed A Alshahir, Omar Alsuhaibani, Asem Beek, Mohammad Alduheim, Ammar Alzahim, Sarah M Alzolaibani, Basem Alhusaini

**Affiliations:** 1 College of Medicine, Taibah University, Medina, SAU; 2 College of Medicine, King Saud Bin Abdulaziz University for Health Sciences, Riyadh, SAU; 3 College of Medicine, King Saud University, Riyadh, SAU; 4 College of Medicine, University of Hail College of Medicine, Hail, SAU; 5 Department of Plastic and Reconstructive Surgery, King Fahad General Hospital, Medina, SAU

**Keywords:** vascular injury, trauma, soft tissue injury, fracture, etiology, compartment syndrome, hand

## Abstract

Hand compartment syndrome is a limb-threatening emergency. Although it is a relatively uncommon condition, early diagnosis, and urgent fasciotomy can prevent irreversible ischemia, myonecrosis, nerve dysfunction, and subsequently permanent loss of hand functions. The occurrence of hand compartment syndrome is relatively infrequent, resulting in a limited amount of literature on its causes. As a result, we conducted a systematic review to provide the most comprehensive data regarding the etiology of traumatic hand compartment syndrome.

This systematic review was conducted and reported in light of the Preferred Reporting Items for Systematic Reviews and Meta-Analysis (PRISMA) checklist. We searched among Medline, and EBSCO Database, with no restriction on the dates (last date of the systematic search was done on April 28, 2022). We included all studies containing data regarding traumatic hand compartment syndrome.

A total of 29 articles with 129 patients constituted the basis of this review. The etiology of traumatic hand compartment syndrome was classified into three groups: soft tissue injury-related, fracture-related, and vascular injury-related causes. The most common etiology of hand compartment was related to soft tissue injuries which constituted 86.8% of all etiologies, followed by fracture-related (5.4%), then vascular injury-related (1.5%). Further, burns were the most likely injury to lead to hand compartment syndrome which made up 63.4% of soft-tissue injuries, followed by animal bites (8.9%).

Hand compartment syndrome can be caused by multiple etiologies that affect people of different ages. Therefore, identifying the most prevalent causes can help in early detection of compartment syndrome by frequent assessment of patients that present with the most prevalent causes like burn among soft tissue injuries and metacarpal bone fracture among fractures.

## Introduction and background

Hand compartment syndrome is a limb-threatening emergency. It is defined as elevated pressure within an enclosed osteofascial compartment to the extent where the circulation and tissue perfusion within the compartment is compromised. Although it is a relatively uncommon condition, early diagnosis, and fasciotomy prevent irreversible ischemia, myonecrosis, nerve dysfunction, and subsequently permanent loss of hand functions [1]. Historically, the five Ps - pain, pallor, paresthesia, paralysis, and pulselessness - have been considered diagnostic findings during physical examination of the affected hand [2]. However, the most reliable symptom is persistent and progressive pain that is disproportionate to the underlying cause and worsens with passive movement [3,4]. Even though the diagnosis of compartment syndrome is usually made based on clinical signs and symptoms, objective measurement of the intercompartmental pressure can confirm the diagnosis in clinically suspected compartment syndrome, especially in unconscious or sedated patients where the diagnosis can be challenging [1]. In spite of the fact that compartment syndrome comprises the affected limb, however, the patient’s life can be threatened. Prolonged muscle ischemia will lead to the release of intracellular contents of myocytes such as myoglobin leading to acute kidney injury. Hyperkalemia, which also results from myocyte necrosis, can cause cardiac conductive abnormalities which may ultimately lead to cardiac arrest [5]. Compartment syndrome results from an increase in intercompartmental pressure; this can arise from a decrease in compartmental size such as tight dressing, or localized external pressure. Also, it results from an increase in compartmental content such as trauma, bleeding, extravasation, and insect bite [6]. Trauma with a concomitant fracture is the most common cause of compartment syndrome in general [7]. Since hand compartment syndrome has a low incidence, the available literature regarding the etiology of hand compartment syndrome mostly contains case reports, and small case series, with only a few large studies [8-17]. In this systematic review, we assess the etiology of traumatic hand compartment syndrome.

## Review

Materials and methods

**Figure 1:**
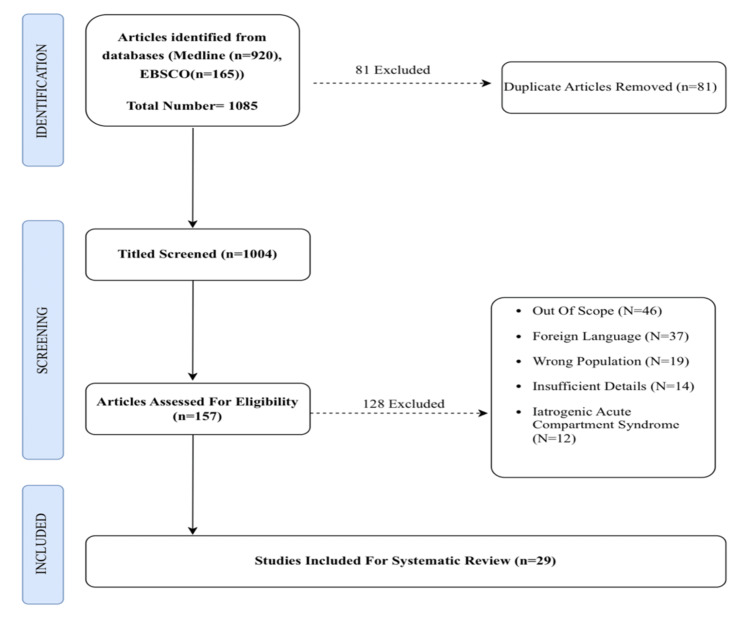
PRISMA flowchart

This systematic review was conducted and reported in light of the Preferred Reporting Items for Systematic Reviews and Meta-Analysis (PRISMA) checklist (Figure 1).

Eligibility Criteria

All clinical studies included patients older than 18 years who presented with acute compartment syndrome (ACS) of the hand related to traumatic injuries and were eligible for systematic review. Clinical studies that included patients with non-traumatic causes of acute compartment syndrome, such as exertional, pathological, chronic, or iatrogenic, were excluded. Studies that involved patients with acute compartment syndrome occurring in regions other than the hand were also excluded. Non-human studies, overlapping data, review articles, case reports, guidelines, comments, errata, letters, book chapters, editorials, and meeting abstracts were not considered.

The screening of titles, abstracts, and full texts of relevant articles was carried out blindly by two authors to identify eligible studies for data extraction. Controversial findings were resolved through discussion with a third author. The flow chart provided details of the screening and eligibility processes.

Search Strategy

We searched among Medline, and EBSCO Database, with no restriction on the date, using these keywords: Compartment syndrome AND Hand OR thenar OR hypothenar OR Dorsal interossei OR Volar interossei OR interosseous compartment OR Adductor pollicis compartment OR mid-palm compartments. The last date of the systematic search was done on April 28, 2022. We reviewed the included studies' reference lists for potentially missed articles that fit our criteria.

Study Selection and Extraction of Data

Eight authors carried out eligibility screening for titles and abstracts. Then full-text assessment and data extraction from eligible articles were carried out independently and duplicated. Finally, any disagreement was settled by discussion or decision of a third author.

Results

A total of 157 articles were retrieved, and only 29 of them met the inclusion criteria. A total of 128 reports were excluded, 46 of them were out of scope, 37 of them were in foreign languages, 12 iatrogenic, 19 wrong population, and 14 articles had insufficient details. Thus, a total of 29 articles with 129 patients constituted the basis of this review [9,11,13,15,18-42].

We classified the etiology of the hand ACS into three groups according to the affected anatomical structure that caused the compartment syndrome. These are soft tissue injury-related, fracture-related, and vascular injury-related causes.

Soft Tissue Injury-Related ACS

Twenty articles provided relevant data about 112 patients who developed ACS (86.8% of all patients) after a soft tissue injury [9,11,18-19,21-25,27,30,32-33,35-40,42]. Four of these identified burn as the most prevalent cause of soft tissue trauma-related ACS, leading up 71 cases (63.4% burned patients) of soft tissue injured patients [30,32,40,42], followed by seven articles reported animal bites making up to 10 cases (8.9%) of soft tissue injured patients [19,22-25,38,39]. Two articles did not specify the extent of the trauma, reporting generic soft tissue injuries in 21 patients as the cause of ACS (Table 1) [9,21].

**Table 1:** Soft tissue-related acute compartment syndrome.

N	Name of soft tissue injury	Number out of 113 soft tissue injury patients	Percentage out of 113 soft tissue injury patients
1	Washing machine roller (Crush) [18]	1	0.9%
2	(Laid on hand after drug abuse, so caused by the pressure of body on hand) [18]	3	2.7%
3	Heroin (Narcotic Overdose) [11]	1	0.9%
4	Not reported soft tissue injury [9,21]	21	18.6%
5	Crush [27,35-36]	3	2.7%
6	Narcotic Overdose [33,38]	2	1.8%
7	Animal bite [19,22-25,37,39]	10	8.8%
8	Burn [30,32,40,42]	71	62.8%

Fracture-Related ACS

Six articles provided relevant data about seven fractured patients who developed ACS (5.4% of all patients) [13,15,26,28,31,40]. Two of these identified 1st metacarpal bone being the most prevalent site of fracture leading to up to two cases (28.8%) of fractured patients causing ACS [15,28], followed by other types of fracture which represent one case for each type - 2nd, 3rd, and 4th metacarpal bones fracture [26], 3rd metacarpal and triquetrum [15], distal radius and ulnar styloid fracture [13], distal radius, scaphoid, triquetral, and capitate bones [31] and dislocation of hamate with hook fracture (Table 2) [41].

**Table 2:** Fracture-related acute compartment syndrome.

N	Type of fracture	Number out of 7 fractured patients	Percentage out of 7 fractured patients
1	2nd, 3rd, and 4th metacarpal bones fracture [26]	1	14.3%
2	1st metacarpal bone [15,28]	2	28.8%
4	3rd metacarpal and triquetrum [15]	1	14.3%
5	Distal radius and ulnar styloid [13]	1	14.3%
6	Distal radius, scaphoid, triquetral, and capitate bones [31]	1	14.3%
7	Dislocation of hamate with hook fracture [41]	1	14.3%

Vascular Injury-Related ACS

Two articles related ACS to vascular injuries in two patients (1.5% of all patients) [18,29]. One paper [18] reported one patient developed ACS after brachial artery injuries in 50% of ACS cases secondary to vascular injury and one article also reported one patient with ACS, developed after ulnar artery injury (Table 3) [29].

**Table 3:** Vascular injury-related acute compartment syndrome

N	Name of the vessel	Number of patients out of 2 patients with vascular injury	Percentage out of 2 patients with vascular injury
1	Brachial artery [18]	1	50%
2	Ulnar artery [29]	1	50%

One article was suitable for being included in two of the three groups, which were included in soft tissue injury-related and vascular injury-related groups [18], and two articles did not specify the anatomical structure in seven cases as the cause of ACS [33,34].

Diagnosis of ACS is made based on clinical findings and intercompartmental pressure measuring in 46 cases (35.7%) [11,15,19,25,30,31,38], on the other hand, 45 cases diagnosed based on clinical findings only (34.9% of all cases) [18,22-24,26,27-30,32,34,35-37,39,41-42]. The remaining cases did not specify the method of diagnosis in 38 cases (29.5%) [9,20-21,34].

Discussion

The incidence of traumatic hand compartment syndrome is a rare entity in the literature. Throughout our systematic review of the literature, only 129 cases of traumatic hand compartment syndrome were identified. Nevertheless, this systematic review aimed to reveal the etiologies of trauma-related hand compartment syndrome. The study assessed 129 patients and revealed that the most common etiology was related to soft-tissue injury with burns being the most common cause of soft-tissue-related hand compartment syndrome. Fractures were the second most common cause and followed by vascular injury. In the majority of reviewed articles, the percentages were similar between the included studies with regard to the method of diagnosing hand compartment syndrome.

In this review, soft-tissue injury affected 112 patients which constituted 86.8% of all hand compartment etiologies. Burns were the most likely injury to lead to hand compartment syndrome which made up 63.4% of soft-tissue injuries. The cause of burn-related compartment syndrome may be attributable to loss of skin elasticity, or increasing voluminous edema which can lead to increasing interstitial pressure and leading to compartment syndrome, particularly when patients are under aggressive fluid resuscitation [43]. Additionally, burn-related compartment syndrome may be difficult to manage and diagnose. Usually, patients with burns present with severe pain and require high doses of sedation and analgesics which all together make the usual sign and symptoms of compartment syndrome difficult to collect [44]. Hence, a surgeon’s due diligence is needed when dealing with burn-related compartment syndrome.

Fractures are a rare cause of hand compartment syndrome. Following our review of the literature, only seven patients who developed compartment syndrome following a fracture were found, with no consistency in the mechanism of injury or type of fracture. In contrast, previous reviews found that fractures were the most common cause of foot, leg, and forearm compartment syndrome [45-48].

Of the 129 included cases in this study, 46 (35.7%) cases were diagnosed using clinical judgment supplemented with intercompartmental pressure measurement, while 45 (34.9%) cases were diagnosed using only the clinical presentation of patients. The early diagnosis and management of compartment syndrome are crucial to avoid dreadful complications. Compartment syndrome is mostly a clinical diagnosis. Moreover, the diagnosis is usually made over time as the signs and symptoms progress and eventually gives a clear picture of the presentation. The classical “P’s” of compartment syndrome which are pain, paresthesia, paralysis, pallor, and pulselessness have been described by McQueen et al. previously to aid in the diagnosis [49]. However, certain situations require the supplementation of intercompartmental measurements such as obtunded patients or intubated patients as well as patients who are in severe pain or sedition, in which the common signs and symptoms of compartment syndrome cannot be assessed adequately [50]. The current study showed similar percentages between the included studies with regard to the methods of diagnosing hand compartment syndrome. This demonstrates that when the clinical presentation is not adequate to diagnose compartment syndrome, intercompartmental pressure measurement should be used to aid in the diagnosis.

Compartment syndrome is a dreadful condition with deadly complications such as gangrene, nerve damage, and rhabdomyolysis if not treated in a timely manner [51]. Hence, this review was conducted to increase the knowledge of treating physicians regarding the various traumatic etiologies of hand compartment syndrome in the literature.

This review faced several limitations. First was the high number of included case reports series. Second, we were unable to obtain a holistic view regarding the different treatment methods option and which was shown to be favorable because of the incomplete data pooled from the studies. Another limitation was the low number of cases in certain etiologies which could inflate the percentages, therefore, looking at the number of cases rather than the percentages to which an etiology represents would be more favorable.

## Conclusions

Hand ACS can be caused by multiple etiologies that affect people of different ages. Therefore, identifying the most prevalent causes can help in early detection of ACS by frequent assessment of patients that present with the most prevalent causes like burn among soft tissue injuries and metacarpal bone fracture among fractures. Intracompartmental measurement is required to diagnose intubated or obtunded patients as well as patients who are under sedation as classical manifestations of compartment syndrome cannot be identified adequately.
